# Structural basis for intra- and intermolecular interactions on RAD9 subunit of 9-1-1 checkpoint clamp implies functional 9-1-1 regulation by RHINO

**DOI:** 10.1016/j.jbc.2024.105751

**Published:** 2024-02-13

**Authors:** Kodai Hara, Kensuke Tatsukawa, Kiho Nagata, Nao Iida, Asami Hishiki, Eiji Ohashi, Hiroshi Hashimoto

**Affiliations:** 1 School of Pharmaceutical Sciences, University of Shizuoka, Shizuoka, Japan; 2 Graduate School of Systems Life Sciences, Kyushu University, Fukuoka, Japan; 3 Faculty of Science, Department of Biology, Kyushu University, Fukuoka, Japan; 4 Nagahama Institute of Bio-Science and Technology, Nagahama, Shiga, Japan

**Keywords:** DNA clamp, 9-1-1, RAD9, RHINO, DNA damage checkpoint, protein-protein interaction, crystal structure

## Abstract

Eukaryotic DNA clamp is a trimeric protein featuring a toroidal ring structure that binds DNA on the inside of the ring and multiple proteins involved in DNA transactions on the outside. Eukaryotes have two types of DNA clamps: the replication clamp PCNA and the checkpoint clamp RAD9-RAD1-HUS1 (9-1-1). 9-1-1 activates the ATR-CHK1 pathway in DNA damage checkpoint, regulating cell cycle progression. Structure of 9-1-1 consists of two moieties: a hetero-trimeric ring formed by PCNA-like domains of three subunits and an intrinsically disordered C-terminal region of the RAD9 subunit, called RAD9 C-tail. The RAD9 C-tail interacts with the 9-1-1 ring and disrupts the interaction between 9-1-1 and DNA, suggesting a negative regulatory role for this intramolecular interaction. In contrast, RHINO, a 9-1-1 binding protein, interacts with both RAD1 and RAD9 subunits, positively regulating checkpoint activation by 9-1-1. This study presents a biochemical and structural analysis of intra- and inter-molecular interactions on the 9-1-1 ring. Biochemical analysis indicates that RAD9 C-tail binds to the hydrophobic pocket on the PCNA-like domain of RAD9, implying that the pocket is involved in multiple protein-protein interactions. The crystal structure of the 9-1-1 ring in complex with a RHINO peptide reveals that RHINO binds to the hydrophobic pocket of RAD9, shedding light on the RAD9-binding motif. Additionally, the study proposes a structural model of the 9-1-1-RHINO quaternary complex. Together, these findings provide functional insights into the intra- and inter-molecular interactions on the front side of RAD9, elucidating the roles of RAD9 C-tail and RHINO in checkpoint activation.

Organisms require genetic stability for survival, necessitating both accuracy of DNA replication and stringent regulation of DNA repair system to minimize heritable mutations. The DNA damage checkpoint serves as a safeguard against genome instability, halting or slowing cell cycle progression in response to DNA damage, including replication stress in a broad sense, to provide time for DNA repair or replication restart (1). The ATR-CHK1 pathway, involving ATR and CHK1 kinases, is a key component of DNA damage checkpoint responses (2, 3, 4). The activation of the ATR-CHK1 pathway is triggered by the accumulation of regions of RPA-coated single-stranded (ss) DNA (5), typically found adjacent to ss/double-stranded (ds) DNA junctions with 5′-ends (5′-junctions), resulting from of fork stalling (6) or ds breaks followed by strand resection (7). Efficient activation of the apical and downstream protein kinases, respectively ATR and CHK1, requires the loading of the 9-1-1 checkpoint clamp onto a 5′-junction by the checkpoint clamp loader, RAD17-RLC (8, 9, 10), along with the involvement of TOPBP1 (11, 12) and RHINO (13, 14).

The 9-1-1 checkpoint clamp, a RAD9-RAD1-HUS1 heterotrimeric complex, shares a structural resemblance with the homotrimeric replication clamp, PCNA (proliferating cell nuclear antigen), featuring a toroidal ring structure (15, 16, 17). Each subunit of the 9-1-1 clamp comprises a PCNA-like domain, which is composed of two structurally similar sub-domains connected by an inter-domain connecting loop (IDC-loop). The PCNA-like domains of the three subunits of 9-1-1 associate in a head-to-tail manner, forming a heterotrimeric ring structure. Among the three subunits of 9-1-1, the RAD9 subunit stands out as the structurally distinctive subunit compared to RAD1, HUS1, and PCNA subunits. RAD9 includes a PCNA-like domain and a C-terminus intrinsically disordered region known as the “C-tail” (RAD9 C-tail). The RAD9 C-tail plays a crucial role in the regulation of the ATR-CHK1 pathway (18). The RAD9 C-tail contains multiple serine and threonine residues subject to phosphorylation (19, 20). Notably, phosphorylation of Ser387 and Ser341 in RAD9 C-tail by casein kinase 2 (CK2) enhances the binding of 9-1-1 to TOPBP1 (21, 22), facilitating the subsequent activation of ATR-CHK1 signaling (23). RAD9 C-tail interferes with the DNA-binding of 9-1-1 (16, 24) and interacts with the toroidal ring of 9-1-1 (9-1-1 ring), whereby the intra-molecular interaction inhibits the interaction between 9-1-1 ring and DNA (24). These suggest that the intramolecular interaction might be a negative regulator of the ATR-CHK1 pathway, preventing undesirable activation. However, it remains unknown to which subunit of 9-1-1 the RAD9 C-tail binds, and the detailed mechanism underlying the intramolecular interaction remains unclear.

RHINO (RAD9, RAD1, HUS1 interacting nuclear orphan) is a conserved protein found in vertebrates, initially identified as a tumor-related protein in humans (25). RHINO interacts with 9-1-1, and this inter-molecular interaction between RHINO and 9-1-1 positively regulates the ATR-CHK1 pathway (13). The crystal structure of 9-1-1 bound to a RHINO peptide has unveiled that the sequence 56-WVSPDF-61 of RHINO interacts with the edge-to-back side of the RAD1 subunit of the 9-1-1 ring (26). It has also been demonstrated that RHINO interacts with both RAD1 and RAD9 subunits of 9-1-1, forming a stoichiometric quaternary complex with 9-1-1 (14). However, it remains unknown which amino acid residues of RHINO are responsible for binding to the RAD9 subunit, and whether RHINO binds to the front or back side of the 9-1-1 ring is also unclear.

Here, we present both biochemical and structural analyses of intra- and intermolecular interactions on the 9-1-1 ring. We show that a hydrophobic pocket on the front side of the RAD9 subunit is implicated in the intramolecular interaction with RAD9 C-tail, suggesting that the hydrophobic pocket is also likely involved in inter-molecular interactions on the 9-1-1. Our crystal structure of 9-1-1 bound to a peptide of RHINO (residues 88–99) reveals that the peptide specifically binds to the hydrophobic pocket on the front side of RAD9. These findings provide a structural basis for understanding both intra- and inter-molecular interactions on the RAD9 subunit, offering insights into the functional roles of RAD9 C-tail and RHINO in checkpoint activation in vertebrates.

## Results

### RAD9 C-tail binds to the hydrophobic pocket on the front side of RAD9

**Figure 1:**
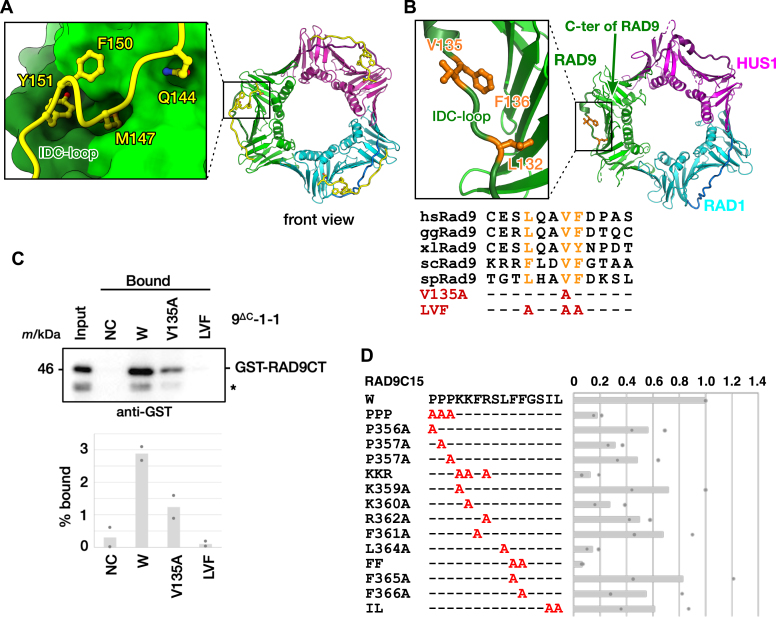
**Binding of RAD9 C-tail to the hydrophobic pocket of RAD9 subunit.***A*, typical interaction of PIP-box with PCNA. The *left panel* shows the interaction between PIP-box and PCNA, a close-up view of a box in the overall structure of human PCNA in complex with the p21 peptide (57) (PDB entry 1AXC). Each subunit of PCNA is shown in *green*, *cyan*, and *magenta*. The IDC-loop of each subunit of PCNA is shown in *darker color*. The p21 peptide bound to PCNA is shown in *yellow*. *B*, structure and alignment of IDC-loop of RAD9. The *left panel* shows the structural detail of the IDC-loop of the RAD9 subunit, a close-up view of a *box* in the overall structure of 9-1-1 ring in the *right panel* (PDB entry 3G65). RAD9, RAD1, and HUS1 subunits of 9-1-1 ring, where the C-tail of RAD9 was truncated (15), are shown in *green*, *cyan*, and *magenta*, respectively. The C-terminus of truncated RAD9 subunit is indicated by a *green arrow*. The IDC-loop of each subunit is shown in *darker color*. Leu132, Val135, and Phe136 in the IDC-loop of RAD9 are colored in *orange*. Amino acid sequence alignment of the IDC-loop of RAD9 is shown in the *lower panel*. hs, *Homo sapience* (Q99638); gg, *Gallus gallus* (Q76F79); xl, *Xenopus laevis* (Q7ZZU5); sc, *Saccharomyces cerevisiae* (Q08949); sp, *Schizosaccharomyces pombe* (P26306). *Dashes* represent the same residue as human RAD9 and “A” represents an alanine substitution. *C*, pull down assay between RAD9 C-tail and 9-1-1 ring. mAG-His-FLAG (NC, negative control), mAG-His-FLAG-tagged wild-type 9-1-1 ring: 9^ΔC^-1-1 (W), or its derivatives: V135A or LVF (L132A/V135A/F136A) mutant was pre-bound to anti-FLAG antibody beads and incubated with GST-tagged RAD9 C-tail (GST-RAD9CT). 0.1% input and 5% bound fractions were analyzed by immunoblotting with anti-GST antibody (*upper*). The percentage of bound GST-RAD9CT was plotted (*lower*). Dots represent individual values from two independent experiments. An *asterisk* (∗) indicates degraded GST-RAD9CT. *D*, contribution of hydrophobic and aromatic residues of RAD9 C-tail to interaction with 9-1-1 ring. GST alone, GST-RAD9C15 that carries residues 356 to 370 of RAD9 wild-type (W), or its derivatives was immobilized on GSH beads and incubated with purified 9^ΔC^-1-1. Bound proteins were analyzed by immunoblotting with anti-RAD1 antibody. Relative intensities of RAD1 signals were quantified, normalized to that of wild-type, and plotted. Dots represent individual values from two independent experiments. Amino acid sequences of RAD9C15 wild-type (W) and its derivatives are shown in *left*. Dashes represent the same residue as wild-type and “A” represents an alanine substitution.

PCNA-binding proteins, such as enzymes and regulatory proteins involved in DNA replication and repair, typically possess a PCNA-binding motif known as a PIP-box (PCNA-interacting protein box) (27, 28), which is defined as QxxΨxxΦΦ, where Q, Ψ, Φ, and x represent glutamine, hydrophobic, aromatic, and any residues, respectively. The toroidal ring structure of the DNA clamp has two sides: the front, alternatively referred to as the C-side (29), and the back. The PIP-box binds to the hydrophobic pocket on the front side of PCNA adjacent to the IDC-loop (Fig. 1*A*) (30). In the case of 9-1-1, which also interacts with multiple partners involved in DNA transactions (31), it has been anticipated, based on the analogy with PCNA, that a hydrophobic pocket adjacent to the IDC-loop on the front side of RAD9 might be utilized for interactions with partner proteins of 9-1-1 (15). However, the mechanisms of these interactions are not well understood.

We hypothesized that the RAD9 C-tail binds to the hydrophobic pocket on the front side of RAD9. To evaluate the hypothesis, we prepared a recombinant protein of 9-1-1 ring that includes a RAD9 subunit with the C-tail truncation (RAD9^ΔC^) and variants of the 9-1-1 ring with substitutions in residues which consist of the hydrophobic pocket of RAD9 (Fig. 1*B*). Specifically, alanine substitution was introduced in Leu132, Val135, and Phe136 in the IDC-loop of RAD9, and the interaction of 9-1-1 ring or its variants with RAD9 C-tail was examined by pull-down assay (Fig. 1*C*). As expected, the V135A substitution of RAD9 resulted in a reduced binding of GST-fused RAD9 C-tail to the 9-1-1 ring; this binding was abolished by the L132A/V135A/F136A triple substitution in the IDC-loop of RAD9 (Fig. 1*C*). This indicates that RAD9 C-tail interacts with the hydrophobic pocket of RAD9 and suggests that van der Waals (vdW) interactions by hydrophobic and/or aromatic residues in RAD9 C-tail are likely crucial for the binding. Indeed, it has been demonstrated that the F365A/F366A double substitution in RAD9 C-tail abolished the interaction with the 9-1-1 ring (24). To gain a more detailed understanding of the interaction within the RAD9 subunit, additional pull-down assays using GST-tagged RAD9 (residues 356–370) as RAD9 C-tail were performed (Fig. 1*D*). Consistent with the previous results, the F365A/F366A double substitution in RAD9 C-tail significantly reduced the binding to the 9-1-1 ring. Notably, the L364A substitution had a pronounced impact on the binding, while the F365A single substitution unexpectedly had a little effect on the binding. Thus, Leu364 and Phe366 in RAD9 C-tail appear to be crucially involved in the binding to the hydrophobic pocket of RAD9 through vdW interactions. Focusing on aromatic residues, the F361A substitution moderately reduced the binding, suggesting that Phe361 is also involved in the binding through vdW interactions. These findings strongly suggest that the hydrophobic pocket of RAD9 accommodates a sequence motif distinct from the PIP-box.

### p21 binds to the hydrophobic pocket of RAD9 independent of PIP-box

**Figure 2:**
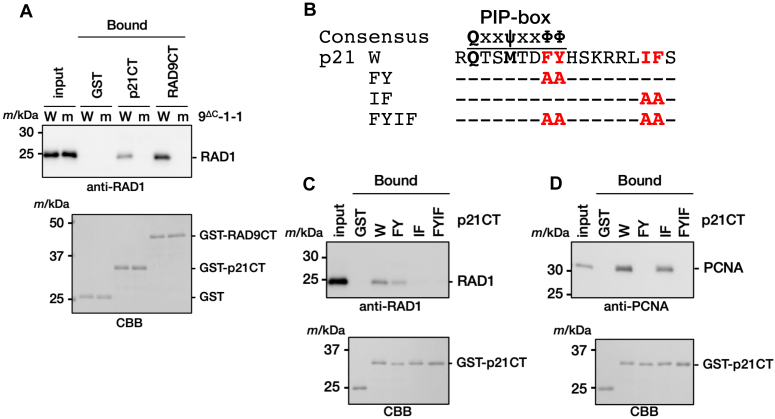
**Interaction between p21 and RAD9 subunit of 9-1-1 ring.***A*, pull down assay between p21 C-terminal region and 9-1-1 ring. 20 pmol of GST alone, GST-tagged p21 C-terminal region (residues 87–164) (GST-p21CT), or GST-RAD9CT was immobilized on GSH beads and incubated with 15 pmol of wild-type 9^ΔC^-1-1 (W) or its LVF mutant (m). Input (2%) and bound (10%) proteins were analyzed by immunoblotting with anti-RAD1 antibody (*upper*). GST, GST-RAD9CT, and GST-p21CT proteins were also visualized by CBB staining (*lower*). *B*, amino acid sequences of p21 wild-type (W) and its mutants used in this study. The PIP-box of p21 is indicated by an overline on the sequence of p21 wild type (W). *Dashes* represent no residue change and “A” represents an alanine substitution. The consensus sequence of the PIP-box is also shown above. *C* and *D*, pull down assay between p21 C-terminal region and 9-1-1 ring or PCNA. 20 pmol of GST alone, GST-p21CT, or its derivatives (FY: F150A/Y151A, IF: I158A/F159A, FYIF: F150A/Y151A/I158A/F159A) was immobilized with GSH beads and incubated with 15 pmol of purified 9^ΔC^-1-1 (*C*) or PCNA (*D*). Input (2%) and bound (10%) proteins were analyzed by immunoblotting with indicated antibodies. GST and GST-p21CT proteins were also visualized by CBB staining.

A previous report showed that p21, the best-known PCNA-binding protein carrying the representative canonical PIP-box in the C-terminal region, FEN1, or p15 binds to 9-1-1, whereas the binding was likely independent of the PIP-box (15). These experimental facts lead us to consider that p21 might also bind to the hydrophobic pocket of RAD9 adjacent to the IDC-loop in a manner similar to that of RAD9 C-tail. To evaluate our consideration, we examined interaction between p21 and 9-1-1 by pull-down assays (Fig. 2). Consistent with the previous report, we observed that the GST-fused C-terminal region of p21 (p21CT) carrying the PIP-box indeed binds to the 9-1-1 ring and a p21CT variant with the F150A/Y151A double substitution in the PIP-box retained the binding (Fig. 2, *A*–*C*). We also confirmed that the recombinant p21CT actually bound to PCNA and the binding was abolished by the F150A/Y151A substitution in the PIP-box (Fig. 2*D*). The 9-1-1 ring variant with the L132A/V135A/F136A triple substitution in the IDC-loop of RAD9 did not bind to p21CT as observed in RAD9 C-tail (Fig. 2*A*). Notably, binding of the 9-1-1 ring to p21CT was also abolished by the I158A/F159A double substitution in p21CT (Fig. 2*C*), while this substitution had a lesser impact on the binding to PCNA (Fig. 2*D*). These results suggest that PIP-box does not bind to RAD9, and an unidentified signature sequence binding to the hydrophobic pocket of RAD9 could be present in RAD9 C-tail and p21CT, and also potentially in other proteins.

### RHINO binds to the hydrophobic pocket on the front side of RAD9

**Figure 3:**
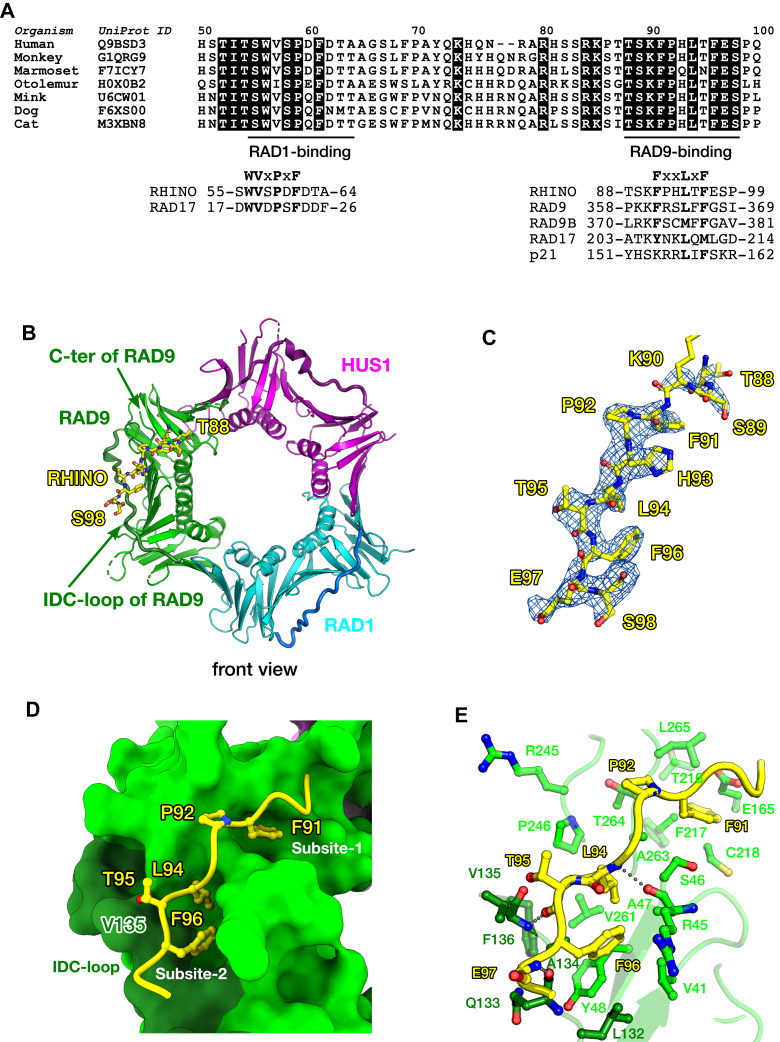
**Structure of 9-1-1 in complex with RAD9-binding region of RHINO.***A*, amino acid sequence alignment of RHINO in vertebrates. Identical residues are highlighted by *black* backgrounds. RAD1 and RAD9-binding regions observed in the crystal structures are indicated by *black lines* below the alignment. RAD1-binding motifs of RHINO (Q9BSD3) and RAD17 (O75943) are shown. RAD9-binding motifs of RHINO (Q9BSD3), RAD9 (Q99638), RAD9B (Q6WBX8), RAD17 (O75943), and p21 (P38936) are also shown. *B*, overall structure of 9-1-1 ring in complex with RAD9-binding region of RHINO. The front view of 9-1-1 is shown as a cartoon model. RAD9, RAD1, and HUS1 subunits of 9-1-1 ring are shown in *green*, *cyan*, and *magenta*, respectively. The IDC-loop of each subunit is shown as a thick tube in a *darker color*. The IDC-loop of RAD9 subunit and the C-terminal residue of RAD9 observed in the crystal structure (Asp267) are indicated by *green arrows*. The RHINO peptide is shown as a stick model in *yellow*. The N-terminal Thr88 and the C-terminal Ser98 of the peptide observed in the crystal structure are labeled in *yellow*. *C*, electron density map of RHINO peptide bound to RAD9. The RHINO peptide is shown in *yellow*. The sigma-a weighted 2m*F*_o_-D*F*_c_ map of the peptide contoured at 1.0 sigma is shown as a *blue* mesh. The amino acid residues of the RHINO peptide are labeled in *yellow*. *D*, interactions of RHINO with the hydrophobic pocket of RAD9. RAD9 is shown as a surface model in *green*. The IDC-loop of RAD9 is shown in *dark green*. Val135 of RAD9 in the IDC-loop is labeled. The RHINO peptide is shown as a cartoon model in *yellow*. The side-chains of Phe91, Pro92, Leu94, Thr95, and Phe96 are shown as *stick* models. *E*, structural details of interaction between RHINO and RAD9. The RHINO peptide is shown as a cartoon and *stick* models as in (*D*). Hydrogen bonds between RHINO and RAD9 are shown as *gray dots*.

RHINO interacts with both RAD1 and RAD9 subunits of 9-1-1 (14). While details of the interaction between RHINO and RAD1 were clarified by the crystal structure of the 9-1-1 ring in complex with a peptide including the sequence 56-WVSPDF-61 of RHINO (26), the RAD9-binding region of RHINO and the mechanism of its interaction with RAD9 are unknown. Assuming that hydrophobic or aromatic residues of RHINO are involved in interactions with RAD9, a conserved region of RHINO (88-TSKFPHLTFESP-99) was considered a candidate peptide binding to the hydrophobic pocket of RAD9 (Fig. 3*A*). Crystallization trials of the 9-1-1 ring in the presence of the RHINO peptide were performed, and crystals suitable for X-ray diffraction study were successfully obtained. Ultimately, the crystal structure of the 9-1-1 ring in complex with the RHINO peptide was determined at 2.81 Å resolution.

The crystal structure reveals that the RHINO peptide binds to the RAD9 subunit of 9-1-1 (Fig. 3, *B* and *C*). In contrast to the RAD1-binding region of RHINO, which binds to the edge-to-back side of the RAD1 subunit (26), the RHINO peptide (residues 88–99) binds to the hydrophobic pocket on the front side of RAD9, across the IDC-loop of RAD9. The hydrophobic pocket consists of two binding sites, subsite-1 and subsite-2 (Fig. 3*D*). RHINO bound to RAD9 adopts an extended conformation (Fig. 3*C*), distinct from the PIP-box that forms a helical conformation upon binding to PCNA. The side-chain of Phe91 of RHINO is accommodated into the subsite-1, and the side-chains of Leu94 and Phe96 of RHINO are accommodated into the subsite-2 (Fig. 3*D*). These side-chains interact with residues in these subsites through vdW contacts. The side-chain of Pro92 of RHINO would also be involved in the interaction with RAD9 (Fig. 3*D*). Hydrogen bonding interactions through main-chains are also observed in the crystal structure, where the main-chains of Leu94, Thr95, and Glu97 of RHINO form hydrogen bonds with the main-chains of Arg45, Val135, and Gln133 of RAD9, respectively (Fig. 3*E*).

Phe91, Leu94, and Phe96 of RHINO correspond to Phe361, Leu364, and Phe366 of RAD9, respectively (Fig. 3*A*), suggesting that interactions of RAD9 C-tail with RAD9 could be similar to those observed in the present structure. Results from biochemical analysis of the interaction of the RAD9 C-tail with the 9-1-1 ring are consistent with those from this structural analysis. As Leu132, Val135, and Phe136 in the IDC-loop of RAD9 are crucially involved to form the subsite-2, it is not surprising that alanine substitutions of these residues had a great impact on the interaction between RAD9 C-tail and 9-1-1 ring (Fig. 1*C*). Phe96 of RHINO, corresponding to Phe366 of RAD9 C-tail, is highly conserved within RAD9-binding proteins and has vdW contacts with Leu132 in the subsite-2 (Fig. 3, *D* and *E*). Consistently, the F366A substitution in RAD9 C-tail drastically reduced the binding to 9-1-1 ring (Fig. 1*D*). Thr95 of RHINO, which is replaced with aromatic or hydrophobic residues in other RAD9-binding proteins (Fig. 3*A*), is closely located to Val135 in the subsite-2 (Fig. 3*D*). This implies that Phe365 in RAD9 C-tail might interact with Val135 by vdW contacts, whereas RAD9 C-tail with the F365A substitution of Phe365 had a little effect on the binding, indicating that the contribution of Phe365 to the binding may be limited (Fig. 1*D*). RAD9B, which is a paralog of RAD9 and predicted to form an alternative 9-1-1 checkpoint clamp (32), also has conserved residues, Phe373, Met376, and Phe378, in its C-tail (Fig. 3*C*). This suggests that the C-tail of RAD9B might be involved in intramolecular interactions, thereby regulating the function of the checkpoint clamp.

The LVF triple substitution in the IDC-loop of RAD9 or the IF double substitution in p21CT abolished the interaction between p21CT and 9-1-1 ring (Fig. 2, *A* and *C*). Based on the crystal structure and sequence conservation, Leu157 and Phe159 of p21 would bind to the subsite-2 on RAD9. Moreover, the hydrophobic side-chain of Ile158 of p21 might contact with Val135 of RAD9.

## Discussion

### RAD9-binding motif

DNA clamp is a universal protein conserved from bacteria to humans and even in some phages. The bacterial homodimeric clamp DnaN, also known as β-clamp, binds to proteins that have a signature sequence termed CBM (clamp binding motif) defined as QL[S/D]LF (33). The PIP-box is the binding motif for PCNA (27, 28), the homo-trimeric replication clamp in eukaryotes. In addition, APIM (AlkB homolog 2 PCNA-interacting motif), [K/R][F/Y/W][L/I/V/A][L/I/V/A][K/R], has been proposed as another PCNA-binding motif distinct from PIP-box (34), whereas both its structure bound to PCNA and its interaction with PCNA are similar to those of PIP-box (35, 36), implying that APIM might be a kind of variant of PIP-box (30). 9-1-1, checkpoint clamp in eukaryotes, is a hetero-trimeric protein that is supposed to orchestrate multiple protein-protein interactions on the 9-1-1 ring through signature sequences specifically binding to each subunit of 9-1-1, RAD9, RAD1, and HUS1. A binding motif for RAD1, WVxPxF, which is found in RHINO (26) and the N-terminal region of RAD17 (37), the large subunit of checkpoint clamp loader RAD17-RLC, has been identified. The RAD1-binding motif has been shown to interact with the edge-to-back side of the RAD1 subunit of the 9-1-1 ring by crystal structures (26, 37). Although RHINO has been shown to interact with not only the RAD1 subunit but also the RAD9 subunit (14), a signature sequence for binding to RAD9 was unidentified. The biochemical and structural analysis revealed the mechanisms underlying intra- and inter-molecular interactions on RAD9, suggesting that the representative sequence for binding to RAD9 would be FxxLxF (Fig. 3*A*). It has been reported that a sequence 205-KYxxL-209 in the AAA+ domain of RAD17 is crucial for the interaction with 9-1-1 (38). Sequence alignment shows that Tyr206, Leu209, and Met211 of RAD17 correspond to Phe91, Leu94, and Phe96 of RHINO, respectively (Fig. 3*A*), suggesting that these side-chains could bind to the hydrophobic pocket of RAD9. During the preparation of this article, a Cryo-EM structure of RAD17-RLC bound to 9-1-1 has been determined at 3.59 Å resolution (39): the structure supports our postulation. Namely, Tyr206, Leu209, and Met211 of RAD17 could bind to the hydrophobic pocket of RAD9. Accordingly, phenylalanine residues in the RAD9-binding motif may be replaced with tyrosine or a hydrophobic residue with some diversity. In the case of p21, the first phenylalanine of the RAD9-binding motif is replaced with Lys154 that unlikely interacts with the subsite-1 due to steric conflict. Such degenerated replacements in binding motifs are also observed in the non-canonical PIP-boxes (30), implying the possibility that further variations of non-canonical RAD9-binding motifs might be found in other proteins.

### Structure of quaternary complex of 9-1-1 and RHINO

**Figure 4:**
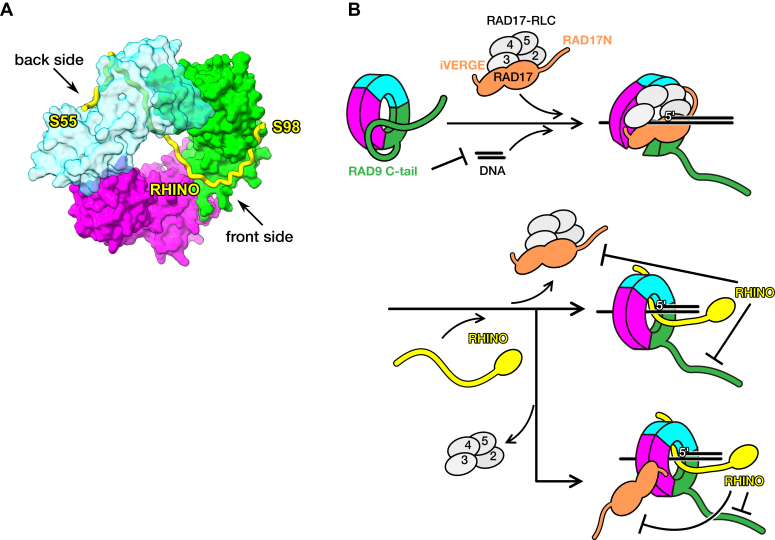
**Implications for checkpoint activation.***A*, plausible model of the 9-1-1-RHINO quaternary complex. RHINO is shown as a *yellow tube*. The coordinates of RHINO residues 55 to 64 extracted from the PDB entry 6J8Y (26) were used to build this model. RAD9, RAD1, and HUS1 are shown as molecular surface models in *green*, *cyan*, and *magenta*, respectively. For clarification, the RAD1 subunit is shown by a semi-transparent model. *B*, potential functions of RAD9 C-tail and RHINO in checkpoint activation. RAD9, RAD1, HUS1, RHINO, and RAD17 are shown in *green*, *cyan*, *magenta*, *yellow*, and *orange*, respectively. RAD17N indicates the N-terminal region of RAD17 that includes RAD1-binding site (37). The acidic C-terminal region of RAD17, iVERGE (47) is also indicated. RFC2, RFC3, RFC4, and RFC5 of RAD17-RLC are shown in *gray* and labeled as 2, 3, 4, and 5, respectively. DNA strands are shown as straight lines in *black*. Before checkpoint activation, 9-1-1 is in a resting state, where RAD9 C-tail binds to RAD9 and covers the central channel of 9-1-1 to inhibit binding of 9-1-1 to DNA (*Upper left*). To activate the checkpoint, 9-1-1 is loaded onto 5′-recessed DNA by RAD17-RLC (*Upper right*). After loading of 9-1-1, RAD17-RLC dissociates from 9-1-1 and RHINO binds to 9-1-1 (*Middle*). In an alternative model, RAD17 binds to 9-1-1 through interaction with HUS1 (*Bottom*).

The crystal structures of the 9-1-1 ring in complex with RHINO peptides reveal that 55 to 64 and 88 to 98 of RHINO bind to the edge-to-back side of the RAD1 subunit and the front side of the RAD9 subunit, respectively. A study showed that 9-1-1 and RHINO form a stoichiometric quaternary complex (14). Assuming that RHINO simultaneously interacts with both RAD1 and RAD9 subunits, a model structure of the quaternary complex is speculated based on our crystal structures, although a structure of RHINO 65–87 residues is missing. To connect the two peptide structures of RHINO with the missing 23 amino acid residues, the polypeptide must pass inside the central channel of 9-1-1, as it is too short to connect the two fragments outside the channel (Fig. 4*A*). While the central channel of DNA clamp has been previously considered for DNA binding, examples of protein binding to the central channel of PCNA or β-clamp were reported (40, 41). For instance, PCNA-associated factor p15 contacts the inside of the central channel of PCNA and passes through the PCNA ring (40). Accordingly, it is proposed that p15 reduces the available sliding surface of PCNA on DNA, thereby modulating sliding on DNA during replication (42). The central channel of the model structure of 9-1-1-RHINO complex has an enough space for ssDNA to freely path through, whereas some interactions with RHINO might be involved for dsDNA. Interestingly, RHINO has conserved lysine and arginine residues in the region between RAD1 and RAD9 binding sites (Fig. 3*A*). These basic residues might be involved in interaction with the phosphate backbone of DNA. Further studies are needed to investigate the interaction between RHINO and 9-1-1 loaded on DNA. In the model structure of the 9-1-1-RHINO complex (Fig. 4*A*), RHINO appears to lock or fasten the ring of 9-1-1, thereby stabilizing the ring structure. This might stimulate checkpoint activation.

### Implications for checkpoint activation

The functional roles of RAD9 C-tail and RHINO in checkpoint activation in vertebrates can be speculated. In *in vitro* experiments, PCNA or β-clamp can bind linear DNA without the clamp loader, because linear DNA can freely access the central channel of the DNA clamp. In contrast, 9-1-1 could not interact with linear DNA, whereas the 9-1-1 ring, in which the RAD9 C-tail is truncated, can bind to linear DNA (16, 24). The present study reveals that RAD9 C-tail binds to the front side of RAD9. Thus, it is plausible that RAD9 C-tail covers the central channel of 9-1-1 as a lid through interactions with the hydrophobic pocket on the front side of RAD9, thereby interfering with the interaction between the central channel and DNA (Fig. 4*B*). The intramolecular interaction by RAD9 C-tail might prevent unfavorable binding of 9-1-1 to DNA and also checkpoint activation. The cryo-EM structures revealed that to load 9-1-1 onto 5′-recessed DNA, RAD17-RLC binds to 9-1-1 and opens the ring structure of 9-1-1 by pulling RAD9 and HUS1 subunits apart (39, 43, 44). The RAD9 C-tail, which covers the central channel of 9-1-1, would be removed from the channel by the interaction between the residues 206-YxxLxM-211 of the RAD17 subunit and the hydrophobic pocket of the RAD9 subunit. After loading of 9-1-1, RAD17-RLC would leave from the 9-1-1 loaded on DNA. Previous studies have revealed that the N-terminal region of RAD17 interacts with the edge-to-back side of RAD1, and the interaction is interfered with by the RAD1-binding region of RHINO (26, 37). RHINO interacts with both RAD1 and RAD9 subunits, and these RHINO-binding sites on RAD1 and RAD9 significantly overlap with the RAD17-binding sites on RAD1 and RAD9, indicating that RHINO might facilitate the leaving of RAD17-RLC from 9-1-1 and prevent re-association of RAD17-RLC with 9-1-1 to avoid unfavorable unloading of 9-1-1. In addition, the binding of RHINO to RAD9 might inhibit the intramolecular interaction within the RAD9 subunit, thereby maintaining the activation. Regarding dissociation of RAD17-RLC, an alternative model has been proposed (39). After loading, the RFC2-RFC3-RFC4-RFC5 (RFC2–5) sub-complex, which is the universal component in clamp loaders, might leave from 9-1-1, and the RAD17 subunit might still be associated with 9-1-1. Day *et al.* (39) state that this model might account for the phosphorylation of RAD17 by the activated ATR (45) and the interaction of RAD17 with NBS1 (46). However, the binding of RHINO to 9-1-1 appears to exclude RAD17 from 9-1-1. Recently, an *in silico* study predicted that the C-terminal acidic region termed iVERGE of RAD17 interacts with HUS1 (47). Therefore, the alternative model might be possible if RAD17 still binds to 9-1-1 through the interaction between iVERGE of RAD17 and the HUS1 subunit, even in the presence of RHINO.

Here, we clarify the structural basis of intra- and inter-molecular interactions on the RAD9 subunit of 9-1-1, implying the functional roles of these interactions in checkpoint activation. We proposed a structural model for 9-1-1-RHINO quaternary complex, where RHINO traversed the central channel of 9-1-1. Our model needs to be validated by further structural and biochemical studies. This is our next priority.

## Experimental procedures

### Expression of GST and GST-fusion proteins

The plasmid expressing GST-tagged RAD9 C-terminal region (GST-RAD9CT) was described previously (24). The plasmids expressing GST-tagged p21 C-terminal region (GST-p21CT) was gifted from Dr Anindya Dutta (The University of Alabama at Birmingham) (48). The plasmids expressing its derivatives were constructed by site-directed mutagenesis using oligonucleotides listed in Table S1. The plasmids expressing GST-tagged RAD9 (residues 356–370) (GST-RAD9C15) or their derivatives were constructed by inserting the annealed oligonucleotides (Table S1) between the BamHI and XhoI sites of pGEX-6P-3 (Cytiva). These plasmids were introduced into an *Escherichia coli* strain, Rosetta 2(DE3)pLysS (Merck). The cells carrying each plasmid were grown in LB medium at 37 °C to OD600 = 0.6, followed by addition of final 0.2 mM IPTG and incubation at 25 °C overnight. The cells were collected, lysed by sonication in Lysis buffer (20 mM Tris-HCl (pH 8.0), 150 mM NaCl, 0.5% NP-40, 1 mM EDTA, 1 mM DTT, 1 mg/ml of lysozyme, 1 mM PMSF, and 20 μg/ml leupeptin) and centrifuged at 7.2 × 10^4^*g* for 30 min at 4 °C. The supernatant was recovered and used for GST pull-down assay.

### GST pull-down assay

The plasmid expressing FLAG-tagged RAD9^ΔC^ L132A/V135A/F136A (LVF) mutant was constructed from pFastBac1-FLAG-RAD9^ΔC^ (24) by site-directed mutagenesis by using oligonucleotides listed in Table S1. FLAG-tagged 9^ΔC^-1-1 (9-1-1 ring) and its LVF mutant used for experiments shown in Figures 1*D* and 2 were produced in High Five insect cells and purified as reported previously (24). PCNA was purified as previously described (49). The cell lysates expressing GST or GST-fusion proteins were incubated with Glutathione-Sepharose 4B (Cytiva) beads for 2 h at 4 °C. The beads were washed four times with buffer H (25 mM HEPES-NaOH (pH 7.8), 150 mM NaCl, 1 mM EDTA, 10% glycerol, 0.01% NP-40, 0.1 mM PMSF, and 2 μg/ml leupeptin). The beads immobilized with GST or GST-fusion proteins were incubated with purified PCNA, FLAG-tagged 9^ΔC^-1-1(W), or its mutant (LVF) for 2 h at 4 °C in 25 μl of buffer H, followed by five washes with the same buffer. The bound proteins were eluted by boiling in SDS sample buffer (50 mM Tris-HCl (pH 6.8), 0.1 M DTT, 2% SDS, 0.05% bromophenol blue, and 10% glycerol), separated by 12.5% SDS-PAGE, and analyzed by immunoblotting using the indicated antibodies. The band intensities were quantified by Image J software (NIH).

### Binding assay of RAD9 C-tail to FLAG-tagged 9^ΔC^-1-1, and its derivatives

For binding assay shown in Figure 1*C*, we constructed the plasmids expressing mAG (monomeric Azami Green)-His-FLAG-tagged RAD9 or its derivatives under the control of EF1 promoter as follows: The BamHI-NotI DNA fragment encoding FLAG-tagged RAD9^ΔC^ (residues 1–272) from pFastBac1-FLAG-RAD9^ΔC^ (24) was transferred between the same sites of pcDNA3. The BamHI-XbaI fragment of the resultant plasmid was inserted between the same sites of pCSII-EF-MCS-mAG-6His-Claspin-3FLAG plasmid (50), replacing the Claspin-3FLAG with FLAG-RAD9. The plasmids expressing its mutants (V135A and LVF) were constructed by site-directed mutagenesis using oligonucleotides listed in Table S1. To construct HUS1 and RAD1 expression plasmids, the *HUS1* or *RAD1* cDNA subcloned into pET20b were amplified with PCR using primer sets, EcoRI-HUS1 and T7 terminator, or EcoRI-RAD1 and T7 terminator, respectively (Table S1). The PCR fragments were digested with EcoRI or EcoRI-XhoI, respectively, and inserted into the corresponding sites of pCSII-EF-MCS Ver. 3-4 (51), resulting in pCSII-EF-HUS1 or pCSII-EF-RAD1, respectively.

These pCSII-EF-based plasmids were used for expression of FLAG-tagged 9^ΔC^-1-1 in HEK293T cells. HEK293T cells were grown in D-MEM supplemented with 10% fetal bovine serum at 37 °C in 5% CO_2_. HEK293T cells were seeded on a 15 cm dish at 6.0 × 10^6^ cells per dish and transfected after 24 h with 4 μg of either FLAG-RAD9^ΔC^, FLAG-RAD9^ΔC^(V135A), or FLAG-RAD9^ΔC^(LVF) along with 6 μg each of HUS1 and RAD1 expression plasmids by using polyethylenimine (Polyscience, Inc) as described previously (51). Cells were collected after 48 h, washed with PBS, suspended in 600 μl of buffer H containing 500 mM NaCl, incubated on ice for 30 min, and added with 600 μl of buffer H without NaCl. The lysates were clarified by ultracentrifugation at 7.2 × 10^4^*g* for 30 min at 4 °C, and the supernatant was recovered. Three-hundred microliters of the lysate were added with 200 μl of buffer H without NaCl and incubated with 5 μl of anti-FLAG antibody beads (Merck) for 1 h on ice. After washing the beads with binding buffer (20 mM Tris-HCl (pH 8.0), 50 mM NaCl, 10% glycerol, 1 mM MgCl_2_, 20 mM β-glycerophosphate, 2 mM Na_3_VO_4_, 0.2 mM NaF, 0.1 mM PMSF, and 2 μg/ml leupeptin), 200 pmol of GST-RAD9CT purified from *E. coli* was added and incubated for 2 h on ice. After washing the beads with the same buffer, the bound fractions were analyzed by CBB staining and immunoblotting with anti-GST antibody.

### Antibodies

The rabbit RAD1 polyclonal antibody was a gift from Dr Katsunori Sugimoto (Rutgers, The State University of New Jersey). The GST and PCNA monoclonal antibodies were purchased from Santa Cruz Biotechnology. The HRP-conjugated goat anti-rabbit IgG and HRP-conjugated goat anti-mouse IgG were purchased from Bio-Rad.

### Crystallization and structure determination

The recombinant human 9-1-1 ring was prepared by a procedure based on a previously reported protocol (26). In brief, the C-terminal truncated RAD9 (residues 1–270), N-terminal His-tag-fused HUS1, and RAD1 were co-expressed by *E. coli* BL21(DE3) with IPTG induction. The 9-1-1 was purified by HiTrap Heparin, HiTrap Q, and HiLoad Superdex200 columns (Cytiva). Purified protein was concentrated, frozen in liquid nitrogen, and stored at 193 K until use. The human RHINO peptide (88-TSKFPHLTFESP-99) was commercially synthesized (TORAY Research Center, Inc). The peptide was dissolved in a buffer composed of 20 mM HEPES-NaOH pH 7.4, 100 mM NaCl, and 5 mM DTT. Crystals suitable for structure determination were obtained by using the 9-1-1 with the triple substitutions F64A/M256A/F266A in RAD1 subunit (26). About 10-fold molar excess of the peptide was incubated with 0.13 mM 9-1-1 at 277 K for overnight. Crystallization was performed by a conventional vapor-diffusion method. Plate-shaped crystals were grown with a reservoir solution composed of 0.2 M potassium iodine and 14% PEG 3350 at 293 K within 2 weeks. Crystals were transferred to a buffer composed of the reservoir solution and 20% ethylene glycol. X-ray diffraction experiments were performed at Photon Factory beamline BL-17A in KEK High Energy Accelerator Research Organization (Tsukuba, Japan). Diffraction data were collected by an Eiger X16M single photon counting detector (DECTRIS, Baden-Daettwil, Switzerland) under N_2_ gas stream at 100 K. Diffraction data were processed with the program XDS (52). The crystal structure was determined at 2.81 Å resolution by the molecular replacement method using the program PHASER (53). The coordinates of 9-1-1 from PDB entry 6J8Y were used as the probe structure in molecular replacement. The electron density of the bound peptide was clearly observed in the difference Fourier map calculated after several rounds of structure refinement by the program PHENIX (54). The structure of RHINO peptide was manually built with the program Coot (55) followed by rounds of model improvement and refinement were continued until convergence. The final electron density of the RHINO peptide is shown in Figure 3*C*. Data collection and refinement statistics are given in Table 1. Atomic coordinates and structure-factor amplitudes were deposited in the Protein Data Bank Japan (PDBj) (PDB entry 8WU8). Figures of protein structures were prepared with the programs PyMOL (Schrödinger, LLC, NY) or ChimeraX (56).

**Table 1:** Data collection and refinement statistics

Data collection	
Space group	*P*2_1_
Cell dimensions	
*a*, *b*, *c* (Å), *β* (°)	71.12, 69.94, 83.95, 97.36
Resolution (Å)	44.45–2.81 (2.96–2.81)
*R*-merge/*R*-meas	0.045 (0.443)/0.063 (0.619)
*I*/σ*I*	10.0 (1.7)
CC half	0.997 (0.660)
Completeness (%)	98.7 (99.8)
No. total/unique reflections	66,834 (10,211)/19,841 (2905)
Multiplicity	3.4 (3.5)
Wilson B-factor (Å^2^)	72.95
Refinement	
Resolution (Å)	35.77–2.81 (2.88–2.81)
No. refined/free reflections	19,825/1982
*R*-work/*R*-free	0.2148 (0.3034)/0.2669 (0.3460)
No. atoms	
9-1-1/RHINO peptide	6080/91
Averaged B-factors (Å^2^)	
9-1-1/RHINO peptide	68.79/82.74
RMS deviations	
Bond lengths (Å)/angles (°)	0.012/0.800
Ramachandran Plot	
Favored (%)	94.5
Allowed (%)	5.5
Outliers (%)	0
PDB entry	8WU8

Values in parentheses are for highest resolution shell.

## Data availability

The coordinates for the 9-1-1 bound to the RHINO peptide and the structure factors are deposited with the Protein Data Bank, accession code 8WU8. All other data will be made available on request.

## Supporting information

This article contains supporting information.

## Conflict of interest

The authors declare that they have no conflicts of interest with the contents of this article.
